# Simulation training on respectful emergency obstetric and neonatal care in north-western Madagascar: a mixed-methods evaluation of an innovative training program

**DOI:** 10.1186/s41077-024-00289-0

**Published:** 2024-05-13

**Authors:** Julie Guérin Benz, Giovanna Stancanelli, Monica Zambruni, Manjary Ramasy Paulin, Habéline Hantavololona, Vonimboahangy Rachel Andrianarisoa, Harolalaina Rakotondrazanany, Begoña Martinez de Tejada Weber, Flavia Rosa Mangeret, Michael R. Reich, Anya Guyer, Caroline Benski

**Affiliations:** 1 Faculty of Medicine, University of Geneva, Geneva, Switzerland; 2 Terre Innovative Healthcare S.R.L, Catania, Italy; 3 Département d’obstétrique, Département de La Femme, de l’enfant et de l’adolescent, Hôpitaux Universitaires de Genève, Geneva, Switzerland; 4 Inspection de La Santé du District, Centre Hospitalier de Référence du District, Ambanja, Madagascar; 5 Safe Motherhood and Family Planning, Département de La Santé Familiale, Ministère de La Santé Publique, Antananarivo, Madagascar; 6 Neonatology and Paediatric Intensive Care Division, Department of Women, Child and Adolescent, University of Geneva, Geneva, Switzerland; 7 Department of Global Health and Population, Harvard T.H. Chan School of Public Health, Boston, MA USA; 8 Boston, USA

**Keywords:** Simulation, Professional training, Emergency obstetric care, Madagascar, Respectful care, Interprofessional collaboration

## Abstract

**Background:**

The rates of maternal and neonatal deaths in Madagascar are among the highest in the world. In response to a request for additional training from obstetrical care providers at the Ambanja district hospital in north-eastern Madagascar, a partnership of institutions in Switzerland and Madagascar conducted innovative training on respectful emergency obstetric and newborn care using e-learning and simulation methodologies. The training focused on six topics: pre-eclampsia, physiological childbirth, obstetric maneuvers, postpartum hemorrhage, maternal sepsis, and newborn resuscitation. Cross-cutting themes were interprofessional communication and respectful patient care. Ten experienced trainers participated in an e-training-of-trainers course conducted by the Swiss partners. The newly-trained trainers and Swiss partners then jointly conducted the hybrid remote/in-person training for 11 obstetrical care providers in Ambanja.

**Methods:**

A mixed methods evaluation was conducted of the impact of the training on participants’ knowledge and practices. Trainees’ knowledge was tested before, immediately after, and 6 months after the training. Focus group discussions were conducted to elicit participants’ opinions about the training, including the content and pedagogical methods.

**Results:**

Trainees’ knowledge of the six topics was higher at 6 months (with an average of 71% correct answers) compared to before the training (62%), although it was even higher (83%) immediately after the training. During the focus group discussions, participants highlighted their positive impressions of the training, including its impact on their sense of professional effectiveness. They reported that their interprofessional relationships and focus on respectful care had improved. Simulation, which was a new methodology for the participants native to Madagascar, was appreciated for its engaging and active format, and they enjoyed the hybrid delivery of the training. Participants (including the trainers) expressed a desire for follow-up engagement, including additional training, and improved access to more equipment.

**Conclusion:**

The evaluation showed improvements in trainees’ knowledge and capacity to provide respectful emergency care to pregnant women and newborns across all training topics. The hybrid simulation-based training method elicited strong enthusiasm. Significant opportunity exists to expand the use of hybrid onsite/remote simulation-based training to improve obstetrical care and health outcomes for women and newborns in Madagascar and elsewhere.

**Supplementary Information:**

The online version contains supplementary material available at 10.1186/s41077-024-00289-0.

## Background

Using simulation techniques during training has proven benefits for improving healthcare workers’ clinical skills, team communication, and interprofessional skills [1–4]. Components of effective simulation include: conducting local unit-based team training; utilizing realistic mannequins; creating opportunities to learn and practice new skills without endangering vulnerable patients; and following up after the training to support trainees to provide patients with the best possible care [5]. Simulation is particularly useful when preparing healthcare workers to provide emergency care, when teams must operate rapidly, smoothly, and cooperatively in those situations to provide high-quality life-saving patient care. Simulation is therefore highly relevant when training providers to attend to women during childbirth [6–8].

Simulation-based training is not common in Madagascar, an island nation off the south-eastern coast of Africa, in either the pre-service or continuing education of health workers [9]. However, simulation-based training has significant potential to have a positive impact in Madagascar, which faces major challenges in preparing and supporting health care providers to deliver high-quality patient-centered health care in many areas, including obstetrics. This paper presents results from an evaluation of an innovative simulation-based training on respectful emergency obstetric and neonatal care that was piloted in Ambanja, Madagascar, in 2021–2022.

### National context

Madagascar is the fourth-poorest country in the world [10]. The effects of poverty and other development challenges are evident in statistics related to childbirth: in 2017, the country’s maternal mortality ratio was 335 deaths per 100,000 live births [11] and the neonatal death rate was 24 deaths per 1000 live births in 2021 [12]. Access to timely and quality obstetrical care is limited: in the northern regions of the country, 60% of women deliver at home and only 46% of deliveries are assisted by a skilled birth attendant [13]. Furthermore, women who deliver at hospitals often do not get quality emergency care, particularly in remote districts.

One obstacle to access to high-quality obstetrical care is the limited training available to health care providers [1, 9, 14, 15]. Pre-professional training for obstetrical staff in Madagascar typically uses classical pedagogical methods. Continuing education opportunities for healthcare workers in Madagascar are both intermittent and ad hoc. The continuing education programs that do exist tend to target either primary care providers working in rural facilities or specialists working at hospitals and universities in major urban centers. Providers who work in peripheral districts’ hospitals have few opportunities to receive refresher training and updated skills development. For example, of the 499 facilities in the country that offer maternity services, only 19 underwent continuing education in Emergency Obstetric and Neonatal Care (EmONC) in the annual report of 2021–2022 [16–19]. However, when obstetric emergencies occur in rural areas and small urban areas, they are referred to the district-level hospitals [18, 20, 21].

### Local context

In the Diana region, located in the far northwest, none of the 15 facilities providing maternity services had been trained in updated EmONC methods. The training described in this paper was offered at one of these facilities, a district referral hospital called the Centre Hospitalier de Référence du District d’Ambanja (CHRD). CHRD is located in the urban center of Ambanja district, which is located over 800 km from the nation’s capital Antananarivo. According to the 2018 census, Ambanja district had a population of almost 237,000 people spread among 18 communes. About ten percent of the district’s population lives in the city of Ambanja. The district has 44 primary health care centers, which refer complicated and emergency cases to CHRD [22].

CHRD conducted more than 1000 deliveries in 2022, approximately 30% of which were Caesarean sections. Between January and September of 2022, nine women and 74 newborns died at CHRD. The hospital’s rates of both maternal and neonatal deaths are more than twice the national rates; while hospital mortality rates are typically higher than national rates due to the referral of complicated cases to hospitals, CHRD’s figures are still highly concerning.

### Establishing a partnership and adapting the training to hybrid delivery

The obstetrical staff at CHRD are committed to providing quality care despite the difficult environment and limited resources. In 2019, four institutions created a partnership in order to respond to a request from CHRD for training in emergency obstetrical care [23]. Two partners were based in Madagascar (CHRD and the Ministry of Health (MOH)) and two were based in Switzerland (Hôpitaux Universitaires de Genève (HUG) and the non-governmental organization Enfants du Monde). Terre Innovative, an Italian social enterprise, provided technical input, as did the World Health Organization (WHO) and United Nations Population Fund (UNFPA), among other agencies. Funding for the training was provided by the ESTHER Switzerland program.

The partners designed the training to address the top 6 direct causes of maternal and neonatal mortality in Madagascar: post-partum hemorrhage, pre-eclampsia/eclampsia, management of labor, obstetric maneuvres for dystocic deliveries, maternal sepsis, and neonatal resuscitation [24]. Simulation was adopted as a key pedagogical method based on the experiences of the Swiss team members, who planned to travel to Madagascar to conduct the training in person.

After the training was initially planned, however, it had to be radically revised in order to adapt to the limitations created by the onset of the global COVID-19 pandemic in 2020. The most obvious challenge was that the Swiss partners could not travel to Madagascar to deliver the training [1, 17, 25, 26]. Other challenges included changing institutional priorities among the partners (as they pivoted to addressing COVID-19) and COVID-19-related supply chain interruptions [27]. The lengthy process of revising the training required all partners to communicate intensively and to be highly flexible. To adapt the training to the new constraints, a training-of-trainers (TOT) component was added to the original plan in order to build the capacity of Malagasy trainers who could participate in person. Various technological tools and platforms—primarily Zoom (Zoom Video Communications, inc, version:5.15.7, www.zoom.us), Moodle (2023 MOODLE UNIGE, Université de Genève, www.moodle.org) and WhatsApp Desktop (2023 © WhatsApp LLC, version 2.2703.10, www.whatsapp.com)— were introduced to enable remote participation by Swiss and Antananarivo-based trainers.

## Implementing the simulation-based training on respectful emergency obstetric and neonatal care

The partnership implemented the training in three phases beginning in June 2021, shown in Table 1.

**Table 1:** Implementation phases of the training program, including key activities, dates, participants, and locations

Phase	Dates	Activity	Locations of participating individuals
Phase 1	15–18 June 2021	Remote TOT for 10 MOH trainers (five doctors, four midwives, and one nurse)	TOT trainees were located in Antananarivo Trainers were located in Geneva
*Evaluation*	*19*–*25 July 2021*	*Baseline data collection on CHRD HR, infrastructure, material, adherence to guidelines, management of labor* *Methods: interviews, direct observation, WHO assessment checklist*	
Phase 2	23–25 July 2021	Didactic training sessions on respectful emergency obstetric and neonatal care	Trainees were located in Ambanja One trainer travelled to Antananarivo 4 remote trainers were located in Antananarivo and 3 in Europe
*Evaluation*	*23*–*25 July 2021*	*Written tests of knowledge administered to all trainees before training*	
Phase 3	31 August–8 September 2021	Group 1: simulation-based training	Trainees were located in Ambanja 6 trainers travelled to Ambanja 4 remote trainers were located in Geneva
Phase 3	13–21 September 2021	Group 2: simulation-based training	Trainees were located in Ambanja 6 trainers travelled to Ambanja 4 remote trainers were located in Geneva
*Evaluation*	*8*–*21 September 2021*	*Written tests of knowledge administered after training*	
Dissemination	24 November 2021	Workshop conducted on Zoom to provide feedback on preliminary results and discuss further partnership	Representatives of WHO, MOH, HUG, CHRD and EDM
*Evaluation*	*6*–*7 April 2022*	*Written tests of knowledge re-administered*	*Trainers participated/observed from Antananarivo, Geneva*
*Evaluation*	*6*–*7 July 2022*	*Focus group discussions with training participants for qualitative evaluation*	*Trainees located in Ambanja* *Facilitator from Switzerland travelled to Ambanja* *Trainers participated/observed from Antananarivo, Geneva*
*Evaluation*	*Ongoing*	*Analysis of evaluation data and writing up results*	

The first phase was the training of trainers (TOT) session, held via Zoom in June 2021 for ten trainers native to Madagascar and selected by the MOH. The TOT course introduced simulation as a pedagogical methodology, including how to conduct pre- and post-briefing processes [5, 8, 27, 28]. It also included reviews of national guidelines on the six topics and best practices for emergency care. The Swiss team used the TOT as an opportunity to practice effectively transmitting information and communicating with trainees remotely.

The CHRD obstetrical staff underwent training during Phases 2 (e-learning sessions) and 3 (simulation sessions) in the following months. Both phases were delivered using a hybrid model that combined on-site and remote training delivery. CHRD provided a dedicated room for the training, which was equipped with internet access, six laptops, six tablets, a conference phone, several smartphones, a projector, and a screen. The trainees and some of the MOH trainers met in person at CHRD. The use of the Internet and technology enabled other trainers in Antananarivo and Switzerland to participate and observe remotely using Moodle and Zoom as communication platforms.

During phase 2, trainers delivered didactic presentations on the six topics and on the management of COVID-19 in maternal care; some topics were presented by an on-site trainer and the rest by remote trainers. The twelve hours of didactic sessions were delivered over the course of 3 days. The sessions were conducted in French, recorded, and uploaded to Moodle so the participants could review them if needed.

Phase 3, which took place a month after phase 2, comprised a series of six facilitated simulation and debriefing sessions on the six topics. The objective of the simulations was to build the capacity of obstetric care teams to work together effectively to provide quality respectful maternal and neonatal care in cases of obstetric emergency. Training on respectful care tends to improve the quality of care and avoid abuses provided by nursing staff in the maternity ward, in particular violence during childbirth or the lack of explanation of the maternal and neonatal medical procedures undertaken [29, 30]. The trainings were conducted by three on-site MOH trainers and other trainers participating remotely from Switzerland. During this phase, the 11 trainees were divided into two groups. Each group participated in an 8-day course (one introductory day, 1 day of simulation for each of the six topics, and a final day for review and evaluation).

A 2-h training session took place on each day of the course, on the participants’ working days. Sessions began with a 10-min pre-briefing, after which the on-site trainers simulated the obstetric emergency using a semi-scripted scenario (see the Appendix for an example). The simulations were conducted in Malagasy. Each session began with a review of our agreed-on principles, including the fact that the training was treated as a “safe container”. Three or four trainees worked as a team to respond to the simulated emergency. For some topics, the trainers acted as the patients; for others, they used the mannequins. Computers and smartphones were used to live-stream the simulation to the remote trainers who were observing and to record the sessions. The remote trainers and one or more on-site participants served as the observers, and a MOH trainer not involved in enacting the scenario provided French translations for the Swiss partners. The Swiss partners also focused on observing body language and interpersonal and team dynamics.

After completing each simulation, the trainees and onsite and remote trainers engaged in extensive structured debriefings and a group evaluation of the team’s actions. Together, they identified any problems and discussed potential strategies for improvement, with a focus on strengthening communication and interaction among staff and attention to the quality of care patients received. Each session ended with a review of the relevant national protocols and standards, as well as time for questions. The aim was twofold: first, to guide the clinical staff through technical aspects of key interventions for obstetric emergencies; and second, to focus on the interpersonal interactions and communication among the clinical staff as they responded to the emergency scenarios. More details on the simulation training are provided in Table 2, which summarizes the relevant elements identified by Cheng et al. (2016) [31] for reporting on healthcare simulation research.

**Table 2:** Key elements of the simulation-based training on respectful emergency obstetric care for CHRD

Element	Sub-element	Training at CHRD
Participant orientation	Orientation to the simulator	One month before the simulation sessions (phase 3), the participants attended the phase 2 didactic training sessions on the six topics with the trainers. On day 1 of the simulation phase, trainers discussed the simulation methodology, including the structure for briefings before and after the scenarios, and the ground rules for the learning exercise. At the outset of each of the six simulation sessions, the trainers presented the scenario.
	Orientation to the environment	The hybrid training was jointly conducted by some trainers in the room with the trainees, and other trainers participating online. The training occurred at the trainees’ workplace in a dedicated training room.
Simulator type	Simulator make and model	2 MamaNatalies (Laerdal) 2 NeoNatalies (Laerdal) Trainers (in person) served as actors
	Simulator functionality	MamaNatalie supports: Deliveries and drills • Normal delivery • Assisted delivery: forceps and vacuum • Breech delivery • Postpartum Hemorrhage NeoNatalie supports: • Oxygen delivery procedures • Suctioning techniques • Positive pressure ventilation • Spontaneous chest rise and fall • Ventilation with bag-valve mask • Closed chest compressions • Auscultate heart sounds • Manual umbilical pulse
Simulation environment	Location	CHRD provided a dedicated training room. The room has enough space to be used for both didactic sessions and for acting out the simulations.
	Equipment	4 mannequins (2 MamaNatalies and 2 NeoNatalies by Laerdal) Internet access, six laptops, six tablets, a conference phone, smartphones, projector, and a screen for remote participants 2 bassinets Mock supplies with realistic packaging
	External stimuli	Normal hospital noise levels
Simulation event/scenario	Event description	Each of the six scenarios was partially scripted (see sample in Appendix).
	Learning objectives	• Understand how the obstetric team can work together more effectively • Understand and implement best practices in treatment of six conditions (post-partum haemorrhage, preeclampsia/eclampsia, maternal sepsis, physiological childbirth, obstetrical maneuvers, neonatal resuscitation))
	Group vs. individual practice	Two groups of 5–6 trainees each (including midwives, nurses, anesthetists and obstetricians)
	Use of adjuncts	n/a
	Facilitator/operator characteristics	10 Malagasy facilitators participated in the Training-of-Trainers sessions. They included five physicians, four midwives and one nurse who were employed by the MOH. The training-of-trainers sessions were conducted by the Swiss team members (all doctors, some with experience with simulation, others with experience with other types of role play and participatory training) In Ambanja, two administrators dealt with all the technological and logistical arrangements, including ensuring that the internet connections worked, logistical support for participants and trainers, etc.
	Pilot testing	No
	Actors/confederates/standardized/simulated patients	Simulated patients were played by facilitators (professional midwives who had participated in the training-of-trainers)
Instructional design (for educational interventions)	Duration	8 days of 2-h sessions: Day 1: Overview of the following days Days 2–7: One simulation session each day Day 8: Review of the training, knowledge test, closing Schedule for simulation sessions: Briefing on the simulation scenario (10 min) Simulation (15 min) Debriefing (40 min) Review of national protocols for treatment of each condition (15 min) Questions and discussion (10 min)
	Timing	Twelve hours of didactic training were conducted 1 month before the simulation sessions. The participants took the same knowledge test three times: once before the didactic learning sessions, on the final day of the simulation sessions, and 6 months after the training.
	Frequency/repetitions	Only once
	Clinical variation	Six topics were covered; each was only covered once (per group)
	Standards/assessment	Utilizing Madagascar national standards (MoH)
	Adaptability of intervention	
	Range of difficulty	Emergency obstetric and newborn care scenarios
	Nonsimulation interventions and adjuncts	One month prior to the simulation sessions, the participants received 12 h of didactic training on the six topics and management of COVID-19. These were delivered by a previously trained MOH trainers in-person coming from the capital (trained during the TOT) and the Swiss teams remotely
	Integration	n/a—this was continuing education, not pre-professional training
Feedback and/or debriefing	Source	1–2 participants and onsite trainers not involved in enacting the scenario were designated as observers using checklists on timing, interaction, and technical skills Trainers participating remotely via Zoom
	Duration	Approximately 40 min of debriefing per scenario
	Facilitator presence	Yes, 5 facilitators were present in the room and 1–3 others remotely
	Facilitator characteristics	See above
	Content	Technical topics: post-partum hemorrhage, pre-eclampsia/eclampsia, management of labor, obstetric maneuvres for dystocic deliveries, maternal sepsis, and neonatal resuscitation Other skills: respectful patient care; inter-personal collaboration on the team
	Structure/method	The trainers guided the trainees through a group evaluation of their actions. Together, they identified any problems and discussed potential strategies for improvement, with a focus on strengthening communication and interaction among staff and attention to the quality of care patients received. The sessions ended with reviews of relevant national protocols and standards and a question-and-answer session.
	Timing	Debriefing was conducted immediately after each simulation
	Video	Yes, both Zoom and videos were recorded on smartphones by two trainers in the room. The Zoom videos showed the full scene while the smartphone recordings provided close-ups.
	Scripting	Yes – one sample script is provided (in French) in the Appendix and all scripts are available from the authors.

## Evaluation methods

Evaluation activities were conducted periodically throughout and after the training in order to: assess the impact of the program on participants’ knowledge, skills, and practices when providing maternal and newborn emergency care [32]; document the adaptation of the simulation training methodology and implementation process to the local context; and elicit the participants’ perspectives on simulation as a training methodology, the relevance of the content of the training to their work, and the hybrid delivery approach.

### Quantitative assessment of knowledge acquisition and retention

The main goal of the training was to impart knowledge on six topics and build professional and team skills. In order to measure knowledge acquisition, a 60-question knowledge test was administered to each CHRD trainee at three points: t0 = before the phase 2 didactic sessions; t1 = immediately after the phase 3 simulation sessions; and t2 = 6 months after the simulation training. The test included ten questions on each of the six training topics.

Analysis of the participants’ scores was conducted using Microsoft Excel (version 16.79.2, 2023 Microsoft 365). Each trainee’s scores were calculated at each time point, including topic scores (that combined the ten questions) and an overall score. Scores were then aggregated by training group and for the full group. We compared scores across the three-time points to determine whether participant knowledge increased after the training and whether the knowledge was retained after 6 months.

### Qualitative assessment of respectful care, patient-centered care and training methodology

The qualitative component of the evaluation sought to capture data in two areas. The first area was the trainees’ experiences with interprofessional collaboration and respectful patient care, which the training emphasized as cross-cutting themes. Second, the partnership wanted to elicit the participants’ opinions about both the relevance of the content and the novel training methodologies (particularly the innovative use of technology for remote participation).

Qualitative data on these two themes were collected through focus group discussions (FGDs) held in July 2022, 9 months after phase 3 concluded. The FGDs were semi-structured sessions in which trainees and trainers were invited to comment on several topics. The two training groups had separate FGDs, each conducted by three on-site facilitators (two trainers from Antananarivo and a medical student from HUG). Another trainer participated in one of the participant groups and others observed remotely. The facilitators and observers agreed that the group dynamics during the FGDs were good, without any notable tensions.

The FGDs were conducted in Malagasy and recorded. The two Malagasy facilitators transcribed the sessions and translated each transcript into French. Their translations were then validated by a professional translator. The transcripts were analyzed separately by two authors (JG and CB, both from Switzerland) with input from a third (HH, from Madagascar). The analysts used inductive thematic analysis to code and develop key themes. Codes and themes identified by each author were compared and discussed to create consensus on the findings.

## Results

Eleven Ambanja hospital staff working in obstetric and neonatal care (including doctors, midwives, and a nurse anesthetist) attended the e-learning and simulation training sessions. Nine of the eleven (82%) participants completed all three knowledge tests and participated in the FGDs in July 2022.

### Quantitative results on the impact of the training on clinical knowledge

**Fig. 1:**
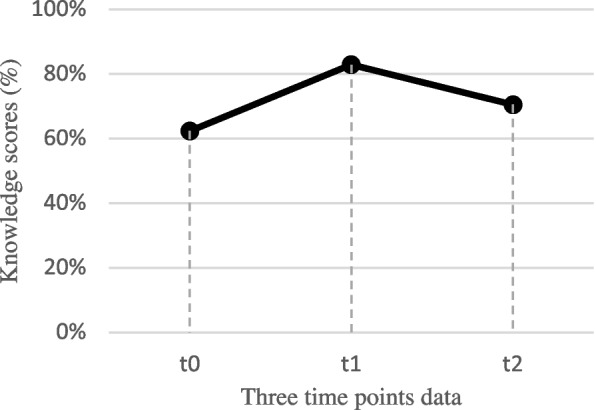
Overall average score at each time point

Trainees completed the knowledge test three times to track their acquisition and retention of knowledge over time. The scores were analyzed both as overall scores and by topic. Figure 1 and Table 3 show the average overall level of knowledge at each time point. Knowledge increased from t0 (62%) to t1 (83%). It then decreased at t2 (71%) although not back to t0 levels. This pattern was similar for all six themes. The results after 6 months (between t0 and t2) range from a gain of just 2% (on obstetric maneuvres) to 12% (on newborn resuscitation). Despite the small sample size, all time intervals separating key program activities show significant results (i.e., *p* ≤ 0.05 with a 95% confidence interval). Indeed, according to Table 3, the results of the *t*-tests according to the values of the quantitative evaluation according to t0, t1, and t2 are as follows: T1–T0 < 0.001, T2–T1 = 0.001, T2–T0 = 0.008.

**Table 3:** Paired samples *t*-test of average score at each point

					95% CI for mean difference					
Measure 1	Measure 2	*t*	df	*t*-test	Mean Difference	SE difference	Lower	Upper	Cohen’s *d*	SE Cohen’s *d*
T1	- T0	11.551	8	< 0.001	17.444	1.510	13.962	20.927	3.850	0.897
T2	- T1	− 4.837	8	0.001	− 9.889	2.044	− 14.603	− 5.175	− 1.612	0.434
T2	- T0	3.528	8	0.008	7.556	2.142	2.617	12.494	1.176	0.396

**Fig. 2:**
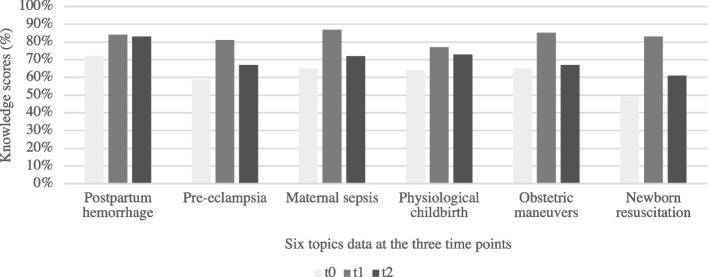
Average scores for each topic at each time point

Figure 2 displays the average result in each of the six topics at the three-time points. The average knowledge scores on the six topics before the training (t0) ranged from a low of 49% (range from 30 to 70%) on newborn resuscitation to a high of 72% (range from 55 to 80%) on postpartum hemorrhage. Immediately after the training (t1), the average level of knowledge was significantly higher, ranging from a low of 77% (range from 50 to 90%) on physiological delivery to a high of 87% (range from 60 to 100%) for maternal sepsis. After 6 months (t2), the knowledge scores had dropped somewhat, although not back to pre-training levels. The average scores at that point ranged from a low of 61% (range from 50 to 80%) on newborn resuscitation to a high of 83% (range from 65 to 100%) on postpartum hemorrhage. The topic scores are ranked in the same order at t0 and t2 (that is, the lowest is resuscitation, and the highest is postpartum hemorrhage). It seems likely, although we do not have data to confirm this, that the providers’ knowledge levels are higher on those problems that they encounter more frequently, which serves to reinforce their learning. Overall, these results suggest that the trainees did learn key concepts and sustained some improvement over time without formal reinforcement. However, they also highlight several opportunities for additional training.

### Differences between the two training groups

**Fig. 3:**
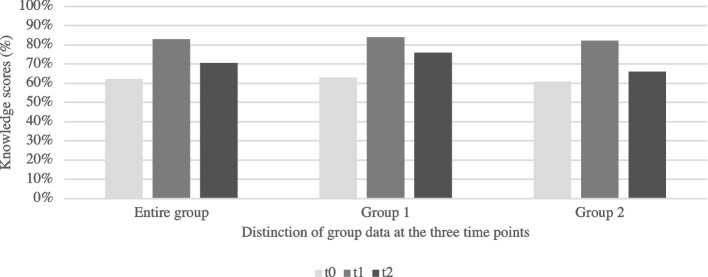
Scores at each time point, disaggregated by participant group

Figure 3 disaggregates the data of the two training groups. The patterns remain similar: there is an overall improvement between t0 and t2, with a peak at t1 immediately following the training.

The available data do not explain the differences between the groups’ retention of knowledge. We can speculate about any number of participant factors (such as previous exposure to the training topics, different experiences in recent clinical practice, or motivation and investment in sustaining learning) or group factors (such as the quality of the simulations and de-briefing sessions, the group dynamics, or the trainers’ levels of engagement in a given session). It may also be a random result of the small sample size in each group.

### Qualitative findings on participant feedback on training methodology and content

Nine months after the training sessions concluded, we held FGDs to elicit the participants’ reflections on five themes: the training experience; simulation and other training methodologies; the use of technology; the impact of the training on their technical and interprofessional skills; and remaining needs. Table 4 presents focus group data representing the connections between the topics and the quotes linked to each, which are further elaborated below.

**Table 4:** Table of qualitative results divided in each theme

Theme	Quote
Experience	“At the beginning there is a little panic, but as we progress it's great” “essential” “excellent” “advantageous” “satisfied” “simulation, humanization of care, prioritization, confidentiality…” “I’m afraid I’ll forget what we studied” “the technique is easily picked up” “Everyone is aware of workload of midwives” “it remained etched in my memory”
Format	“if we extend it, we risk not meeting all the objectives, and if we do it faster, we risk not being able to control it well” “We all participated one by one. Everyone was able to play their assigned role” “the right to ask any question” “experience sharing” “the duration for me is insufficient”
Professional skills	“my working methods have improved especially the preparation and the golden minute” “Training is a kind of recycling of knowledge” “team collaboration has improved” “a friendly atmosphere was created between the patient and me” “I was not able to apply the management of postpartum hemorrhage learned, often the fetuses are already suffering and you have to act quickly”
New technological tools	“It is up to me to make the decision” “we need to assess our understanding of things and also to question freely” “it is a kind of formative evaluation and at the same time we are corrected” “the simulation is really great, but I would also like to do it on a real case” “successful videoconference” “effective method” “it is the simulation that i enjoyed, I loved it”
Needs	“upgrading” “there are still failures, it is human. We ask for more training” “I don’t have enough control” “we also need the peripheries, train them and work together” “we are asking for emergency kits, masks, sterilization equipment, decision tree…” “visual aids” “increase the number of simulation and less theory”

### General reflections on the training experience

The participants’ feedback on the training was generally extremely positive. When discussing the training, participants used terms such as “satisfying” and “excellent”. They reported that the training responded to their felt needs, covered the topics well, and was relevant to the situation they experienced at CHRD. Aspects of the training that participants particularly liked were the simulations, live practice, and videoconferences. The facilitators also noted that the participants demonstrated a strong desire to learn and openness to feedback—as one facilitator said: “We see that you [participants] are eager for knowledge and want to improve in your work.”

None of the participants had previously attended a simulation-based training or even one that required active participation. As one participant said, “Previously, we had trainings…during which we just write and we are given documents. Sometimes we don’t even understand what is in this big book—we just listen.” A trainer echoed this: “As a trainer, what I saw in the past was a little different from what we did, because in general we only do theory. But during this training, it is direct practice….It is true that it is on a mannequin, but you can touch it.” Participants said that following the training, they felt less stress when providing emergency care.

Encountering a new pedagogical method was initially stressful for some participants—one described feeling “a little panic at the beginning” but then noted, “but as we progressed, it was great.” Others said they were initially nervous about “playing themselves” working in stressful situations. However, they quickly understood that the intention of the simulation was to create a “safe environment for training” and they liked being in an “almost real” situation.

Participants also noted that training additional providers, particularly those working at the primary health centers that refer patients to the hospital, could further improve the care they provide to their patients. As one participant said: “Cases of delays [in patients seeking emergency care during deliveries] are very difficult to manage, making the application of our training very difficult….Therefore, we want to spread such training to the [local health center] level [which refers emergencies to CHRD] if possible.” Participants also acknowledged that a single training could not fix all the challenges they face. One said, “There are still failures.…[so] we need to reinforce what we have learned.”

### Specific feedback on the training format and content

Participants considered the contents of the training to be directly relevant to their work. According to one participant, the training has had “a direct impact on [my] professional tasks, with an improvement in working methods.” While the training received mostly positive feedback, participants also shared concerns. They appreciated the format (a didactic learning phase followed by the interactive simulation phase), but three participants suggested holding phase 3 sooner after phase 2. Another common complaint was that the phase 3 was not long enough. Finally, they expressed that it was difficult to participate in the training for part of a day while doing clinical work the rest of the day. One participant explained: “For those who were on duty, it was a bit hard because your concentration is disturbed by the fact that there could be serious cases or emergencies.” Another participant added: “It was a bit harsh [to do the training for a half-day while at work].”

Participants appreciated that training in teams helped them develop interprofessional communication tools, improving their capacity to collaborate. One role-exchange exercise was noted to be particularly beneficial. The participants found it fun to act as a different time of provider and the process promoted convivial group dynamics; more substantively, taking on other roles helped team members feel more comfortable sharing tasks, identify more with their colleagues, and better appreciate the work of others. One participant said: “After the role exchange exercise during the simulation, everyone is aware of the volume of work of a midwife and the importance of everyone’s roles. So [now] the team helps each other.” Improving interprofessional collaboration has enabled CHRD staff to provide and receive feedback. For example, one of the anesthetists noted: “We were able to check what midwives do…[and] we were able to bring ideas and suggestions for improvement.”

Finally, the participants appreciated being able to “ask any kind of questions” without fear of judgment. They enjoyed getting feedback from remote trainers; several participants said this was the most personalized training they had ever done. Communication with the trainers, including those who participated remotely, continued after the training; one participant commented, “We can discuss cases [with them] directly, and it is very advantageous for us.”

### Experience of new pedagogical and technological tools

Both simulation and e-learning represented innovations for the participants. The simulation methodology was perceived by most of the participants as an interesting and effective training tool with much potential. The participants also appreciated the use of e-learning. In part, the use of technology for remote participation was reportedly new. It also offered them the opportunity to learn about newer approaches to health care, such as focusing on quality and the provider-patient relationship.

Participants noted the high quality of the trainers’ instruction and appreciated their focus on improving the performance of the whole team. They felt that both the onsite and remote trainers provided useful feedback, especially when reviewing the simulation exercises. The remote observers were perceived by the participants as non-judgmental: “I have no problem with their presence because they are really correctors. They didn't scold us when we made mistakes. They are real “proof-readers” so no stress.” Trainees said that using videoconferencing worked well to facilitate communication among the Swiss and Malagasy participants.

### Impact of the training on professional skills

This training was developed in response to requests from the providers. They reported feeling that the training had indeed supported them to improve their professional performance. During the FGDs, participants reported that since the trainings they had been utilizing the protocols that had been discussed. One participant stated: “It had a direct impact on our tasks, [and] has led to improvements in our working methods, in the reception of patients in general.” Participants described now feeling “fast” and “efficient” in their work. One participant listed several examples: “It is the GATPA [Gestion Active de la Troisième Période de l'Accouchement, or Active Management of the Third Stage of Childbirth] that I apply in my daily activities. And also the management of eclampsia, because there are many at the CHRD. And also resuscitation. In fact, everything [laughs] [including] postpartum hemorrhage, normal delivery!”.

Participants reported that the training had resulted in improving both their interprofessional communication and their communication with patients. Unprompted, half of the FGD participants mentioned they enjoyed using the SBAR (Situation, Background, Assessment, Recommendation) tool, a technique for conveying clear and concise patient information. One participant said: “The part on SBAR communication…I found interesting, how to call a doctor in the face of an emergency, that we must make a presentation above all. Previously, we used to [just] say: ‘Come Doctor, there is a sick person!” Another participant noted that after the training the obstetrical team had successfully advocated together to get the hospital to purchase a resuscitation table.

Participants did raise some concerns, including about the applicability and sustainability of some training topics. They reported rarely using some of the obstetrical maneuvers and were concerned about losing ground in these areas. As one participant said, “when [the women coming to deliver at CHRD] reach me, it is not possible to practice what we learned during the training because they are often in a critical state. We are obliged to intervene directly in the operating room [to conduct a Caesarean section].” Participants noted that this can be extremely frustrating and upsetting, especially if it results in a death that might have been prevented with earlier intervention.

### Needs and additional prospects

Finally, the FGD participants commented on their remaining training needs and shared ideas for follow-up programming. One participant noted that the training content was “insufficient [if] the objective [is] to accelerate the reduction of maternal and infant mortality.” The participants listed several additional topics on which they wanted simulation-based training, including breech delivery, twins, neonatal resuscitation, and placenta praevia. They also had several suggestions about how to help them retain and reinforce skills learned during the training:

- Improve access to equipment, materials, and medicines in order to enable them to provide quality care in line with their training. Items they mentioned included certain medicines, delivery tables, echocardiography, resuscitation equipment, oxygen, incubators, and blood supplies.
- Offer refresher trainings and other training opportunities. One participant said, “Simulation is really great, but I would like it to be on a real case…where we are assisted.”
- Provide ongoing coaching by the trainers.
- Create posters of updated treatment algorithms and guidelines to be posted as visual reminders.

The participants also had suggestions on areas for improvement when replicating this training, mostly focused on the need for more simulation-based sessions—“less theory, more simulation" was a common refrain.

## Discussion

To our knowledge, this hybrid in-person/remote simulation-based training on respectful obstetric and newborn care was the first of its kind to be offered in Madagascar [22]. Other trainings on EmONC and related topics have been conducted using various methodologies in other locations in Madagascar [16], but not in Ambanja and not at district-level hospitals. Furthermore, globally, few organizations have piloted the use of remote participation in simulation-based trainings [1, 14, 15, 33], and we have not identified others that have been rigorously evaluated. Therefore, our findings are useful on several fronts: assessing the impact of this training; adding to the literature on the use of simulation-based training in continuing education for obstetric health care providers; and, in particular, providing insight into the applicability of hybrid delivery of simulation-based training in Madagascar and other low-resource settings [8, 30, 34].

This evaluation suggests that the training was generally a success in terms of increasing subject knowledge, skills capacity, and interprofessional collaboration among the team at Ambanja District Hospital. From the quantitative data, we found that baseline knowledge (t0) was lowest on the topics of newborn resuscitation and pre-eclampsia. Based on informal observations and discussions we have speculated a few possibilities about low scores obtained by these topics. For instance, the providers tend to overlook newborn resuscitation as an option due to lack of access to equipment, lack of training, and especially their prior experiences with poor prognoses and outcomes for fragile newborns in low-resource settings. Besides, the pre-eclampsia scores were likely low because the providers had been previously trained using outdated protocols, algorithms, and medications and had not received updated training.

The trainees’ knowledge of key facts on all of the six topics peaked immediately after the simulations (at t1), with the greatest increase in knowledge in newborn resuscitation. When we repeated the knowledge test 6 months after the training sessions (t2), we found that knowledge levels on the six topics had fallen after 6 months, but remained higher than before the training. When these results were disaggregated between the two training groups, one showed a notably greater loss of knowledge than the other group. The most concerning finding at t2 was that none of the participants were able to correctly answer at least 75% of the questions on obstetrical maneuvers. One possible explanation is the impossibility of applying the maneuvers learned in most cases because patients who could have been candidates generally already needed an emergency cesarean section when they arrived at the CHRD. At t2, nearly half of the trainees were still able to provide correct answers for at least 75% of the questions on four topics (pre-eclampsia, maternal sepsis, physiological childbirth, and newborn resuscitation) and two-thirds were able to achieve that level on the questions about postpartum hemorrhage.

Taken together, these findings suggest that while the trainees acquired significant new knowledge during the training sessions, this knowledge dissipated over time, particularly on topics that are less frequent occurrences. In addition to specific training events, therefore, we are strategizing with the team in Ambanja about how they can regularly reinforce their knowledge.

The qualitative findings indicated that the trainees liked both the structure and the content of the training. Their interest in the simulation-based methodology was explicitly expressed during the post-training discussions and the FGDs—it was also implied by the trainees’ requests to receive similar trainings on other topics. During the FGDs, the trainees particularly noted the positive effects of the training on interprofessional communication [27, 35, 36]. The hybrid nature of the training was not seen as an impediment to the effectiveness of the simulation methodology. The remote involvement and observation by some of the trainers may in fact have helped the trainees feel comfortable with this type of technology-assisted connection. This in turn has made it easier for them to maintain their connections and smooth communications with the trainers as they continue to provide ongoing mentoring.

## Conclusion

Despite facing challenges, the development, implementation, and evaluation of the hybrid simulation-based training on respectful obstetrical and newborn care that was delivered in 2021 at CHRD proved to be a positive experience for trainers, trainees, and the partnering institutions. We recognize that this instance of hybrid simulation-based training may not be directly replicable elsewhere: it took place during a time when conditions were particularly difficult due to the COVID-19 pandemic and was tailored to a specific set of trainees working together in a single facility. Furthermore, our capacity to do a statistically significant evaluation was limited by the small number of trainees. Despite these limitations, our findings demonstrate that the hybrid simulation-based training methodology can be highly effective at improving topical knowledge, professional skills, and communication among obstetrical care teams working in low-resource settings.

Developing a training that is sufficiently tailored to a local context requires more than technical content. It also requires a strong partnership among the various institutions involved and a collective commitment to tailoring the training to meet local needs. With this training, we have demonstrated the potential of this approach to have a significant positive impact at CHRD, and possibly elsewhere in Madagascar and other countries. The partnership is planning to continue supporting the obstetric team at Ambanja and exploring expansion to other venues in the vicinity. Future activities under development include more hybrid simulation-based trainings (for the original group of trainees on additional topics, and on the original six topics for obstetric team members from the other facilities that refer to CHRD). We are also supporting the team at CHRD as they develop strategies to ensure that their patients receive respectful care, to expand community awareness about the available obstetrical services and patients’ right to respectful care, and to continually maintain and reinforce staff professional capacity.

### Supplementary Information

Supplementary Material 1.

## Data Availability

A sample simulation script (in French and English) is attached. The remaining scripts (in French and Malagasy), training schedule, and evaluation data are available upon request from the authors.

## Abbreviations

CHRD: Centre Hospitalier de Référence du District d’Ambanja
EDM: Enfants du Monde
EmONC: Emergency Obstetric and Neonatal Care
FGD: Focus group discussion
GATPA: Gestion Active de la Troisième Période de l'Accouchement, or Active Management of the Third Stage of Childbirth
HUG: Hôpitaux Universitaires de Genève
MOH: Ministry of Health
SBAR: Situation, Background, Assessment, Recommendation
TOT: Training of Trainers
UNFPA: United Nations Population Fund
WHO: World Health Organization
