# Supplementary material for: Simulation training on respectful emergency obstetric and neonatal care in north-western Madagascar: a mixed-methods evaluation of an innovative training program
Source: Adv Simul (Lond). 2024 May 13;9:18. doi: 10.1186/s41077-024-00289-0 (PMC11092212; doi:10.1186/s41077-024-00289-0)
Supplement: Supplementary file 1 — Supplementary Material 1. [file 41077_2024_289_MOESM1_ESM.zip › Appendix 1_Script for Post-Partum Haemorrhage (PPH) Scenario (in French)_ESM.docx]

**Appendix 1: Script for Post-Partum Haemorrhage (PPH) Scenario (in French)**

(N.B.: Scripts for the other five scenarios are available from the authors)

| **Thème du scénario : Hémorragie post-partum**  Rédacteurs du scénario : A. Chilin / C. Benski | | |
| --- | --- | --- |
| **Objectifs** | | |
| **Médicaux**  Poser le diagnostic de l’hémorragie de la délivrance  Reconnaître l’urgence hémodynamique  Connaître la séquence de la prise en charge  Rechercher les causes les plus fréquentes  Utiliser les médicaments utérotoniques | Gérer les transfusions massives et troubles de la crase  Evoquer une conduite à tenir (planification à moyen terme)  **Collaboration interprofessionnelle** |  |
| **Formateurs, apprenants et rôles** | | |
| **Débriefeur**: Obstétricien  **Facilitateurs :** 1SF  **Briefing / débriefing** :  **Nombre d’apprenants et fonction** : 3 participants actifs, 2 sages-femmes, 1 médecin obstétricien  **Aide supplémentaire** : des observateurs, 1 observateur : délai, 1 observateur : prise en charge médicale | | |
| **Logistique** | | |
| **Matériel :**  Lit  Monitoring maternel (TA, pouls, saturation) ; Manchette à pression  Voies veineuse avec tubulure et robinet à 3 voies  Médicaments : Ocytocine ; Cytotec ; Methérgine ; Nalador ?; Antibiotiques : (céfazoline ou ampicilline IV lente :2gr en dose unique)  Perfusions : Ringer-Lactate ; Chlorure de sodium 0,9% , Macromolécules  Transfusions : sang : donneur universel  Masque O2, sonde urinaire  Gants (à révision) ; pinces à col ; valves ; blouse, champs, compresses  Ballon de Bakri  Moltex plein de sang  Bande élastique autour de la 2^ème^ VVP, retirer la bande pour mimer pose de 2^ème^ voie veineuse  Poupée nouveau-né  Moyens de communication : images de téléphone pour mimer l’appel  Fiches: résultats Hb/ Ht ; Crase ; DA/RU ; utérus | | |
| **Préparation de la patiente standardisée**  Au départ patiente est calme et collaborante. Réponds aux questions dirigées. Manifeste progressivement une fatigue et malaise plus le scénario avance. Perte de connaissance sur signe du Formateur si pas de prise en charge adéquate. | | |
| **Préparation de l’accompagnatrice**  Elément perturbateur: « Ca saigne beaucoup! » « C’est normal tout ce sang » « Mon amie ne se sent pas bien » « Je suis très inquiète pour mon amie ». Va pouvoir être sortie de la pièce sur demande des participants. | | |
| **Briefing** | | |
| Accueil, charte de simulation présentée dans l’introduction générale. | | |
| Explication déroulement de la séance ;  Présentation du matériel :   - Montrer les photos du téléphone - Bien demander d’énoncer haut et fort en cas d’appel au téléphone : QUI j’appelle QUEL degré d’urgence….. - Montrer fiches pour certaines données cliniques à la demande des participants (pertes ; Hb/Ht ; utérus mou-dur ; résultats de la révision des trois étages)   Expliquer qu’il y a du matériel mais que le geste endo-utérin ne sera pas effectué.  Il y a des pancartes avec des informations cliniques ou biologiques qui sont demandées par les participants | | |

| **Briefing du scénario. Distribution des rôles.** |
| --- |
| **Histoire clinique (à lire comme briefing pré-scénario UNIQUEMENT à la sage-femme qui s’occupe de la patiente). Les autres intervenants sortent de la salle**  *On est en salle d’accouchement : on est à 45 minutes d’un accouchement par voie basse.*  *Mme V, patiente de 30 ans, 4G 4P (4 AVB sans particularité)*  *Pour cet accouchement : admise pour un travail spontané.*  *La patiente a accouché rapidement d’un enfant de 4450 g avec un périnée intact. Elle a reçu 5 UI de Syntocinon® aux épaules.*  *Délivrance d’un placenta 15 minutes après l’accouchement, d’aspect complet et normal. Prophylaxie d’atonie au vu de la multiparité avec Syntocinon 20 U sur 6h.*  *Amélioration de la contraction utérine sous ce traitement.*  *Pertes totales estimées à ce moment à* ***400 ml***  *La sage femme quitte la salle : le scénario démarre*  *45 minutes après l’accouchement la patiente signale des pertes de sang plus importantes* ***(« ça coule en bas »)*** *elle a l’impression qu’elle va tomber dans les pommes*  *La sage-femme est rappelée* |
| **Scénario. Durée: 10 minutes** |
| Démarrage scénario dès que appel **« ça coule en bas » :**   - **écoulement manifeste (Affiche avec perte à correspond à 800 cc)** - **utérus  relaché**   **Affiche avec TA à 115/80, puls à 95/min ; saturation 98 %; FR 13 / min**  Depuis appel   - **METTRE AFFICHE : perte 400 cc + 800 cc =1200 cc** - si palpation utérus : **METTRE AFFICHE** : utérus MOU |
| **Evolution du scénario**  PS totales 1200 cc   - **METTRE AFFICHE** même si pas de demandé de quantité de pertes (stress)   **Avec TA à 95/75, puls. à 110/min ; saturation 98 % ; FR 13 / min ; utérus spongieux**  Puis saignements encore de 400 cc   - **METTRE AFFICHE : perte 400 cc + 800 cc + 400cc = 1500 cc** - **TA à 70/40 puls : à 125/min saturation :98% Fr :20/min utérus mou**   **Patiente avec nausée et vomissements (accompagnante : « Mon amie ne se sent pas bien, elle vomit »)**  **Si demandé et réalisé mettre AFFICHE LABORATOIRE : Hb 62 g/ L ; Ht 22 % ; PTT 42 sec ; Fibrinogène 0.9 g/L** |
| **Evolution du scénario**  Amélioration de la situation si prise en charge obstétricale correcte avec   - Remplissage vasculaire avec ringer lactate ou NACL 0,9% (1L en 15 minutes) - Pose d’une sonde de foley - Préparation à une transfusion, vérifier disponibilité du sang, déterminer groupe de la patiente - Administration de utérotoniques de 2^ème^ pallier (Nalador) (+/- Fibrinogène et Cyklokapron) - Massage utérin pour expulser les caillots, compression bi manuelle si saignement important continu - Révision de la filière génitale (vérifier absence de lésion du col ou u vagin) avec RU systématique, installation pour pose de ballon de Bakri |
| **Après tout cela :**  **OPTION A**  **TA à 95/75, puls à 110/min ; saturation 98 % ; FR 13 / min ; utérus spongieux 0%, saignement 0 %**   - **METTRE AFFICHE** : utérus CONTRACTE - **Arrêt du scénario (si stabilisation de la patiente et/ou installation pour Bakri ou planification de embolisation )**   **OPTION B**  Si prise en charge sub optimale : constantes hémodynamiques restent identiques  **TA à 70/40, puls. à 140/min; saturation 90 % ; FR 20/ min¸ utérus toujours atone ; patiente inconsciente**   - Malaise de la patiente avec perte de connaissance - **METTRE AFFICHE** : **patiente inconsciente** - **Arrêt du scénario si**   Evocation de transfert au bloc pour hystérectomie d’hémostase |

| **Observations possibles durant le scénario / actions attendues** |
| --- |
| **Reconnaissance de l’hémorragie du post partum**  monitoring des paramètres vitaux rapprochés  massage utérin  vidange vésicale /pose d’une sonde à demeure  contrôle du placenta  révision des 3 étages (avec antibiothérapie prophylactique)  pose d’une 2^e^ VVP de gros calibre  perfusion de 20 UI de Syntocinon® ou misoprostol par voie sublinguale ou méthylergométrine IM  demande de bilan sanguin  administration de fibrinogène / Cyklokapron®  mesurer le taux d’hémoglobine  si possible oxygénothérapie à haut débit  gestion des contrôles sanguins et transfusions si envisagées ; avertir le Centre de Transfusion  planification de la prise en charge (équipe envisage Bakri ou embolisation) |
| **Débriefing** |
| Définition de l’hémorragie du post partum : prévention par conduite active de la délivrance  Difficulté estimation pertes  Bonne tolérance maternelle au début  Enjeux de morbidité et mortalité maternelle (historique)  Facteurs de risques  Cause les plus fréquentes (4T : tonus ; trauma ; tissus ; thrombine)  Planification, multidisciplinarité et approche par étapes   - Algorythme proposé : à adapter au contexte local - Chronométrage : diminuer les retards de la prise en charge   Etape 1 (0-30 minutes : délai à titre indicatif : timing à adapter à la clinique et à la sévérité)   - Recherche de l’étiologie - Traitement « mécanique » - Réanimation médicale (appel à l’aide/ monitoring / accès veineux / évaluation des pertes (laboratoire) / prévention hypothermie - Administration d’utérotoniques (Syntocinon en perfusion rapide)   Etape 2 (30-60 minutes: délai à titre indicatif : timing à adapter à la clinique et à la sévérité)   - Si échec Syntocinon : autre utérotonique , Methérgine , Cytotec - Compression / Bakri. Poursuite du remplissage. - Stabilisation hémodynamique : Transfusion et stimulation de l’hémostase (fibrinogène / acide tranexamique) - But stabilisation hémostase : Hb>80 g ; Plqt > 50G ; fibrinogène > 1g/L ; TP>50% ; pH > 7.2 ; Temp. >35 °   Etape 3 (>60 minutes: délai à titre indicatif : timing à adapter à la clinique et à la sévérité)   - Prévoir et anticiper le transfert de la patiente vers une structure pour un geste chirurgical |
