# Supplementary material for: Simulation training on respectful emergency obstetric and neonatal care in north-western Madagascar: a mixed-methods evaluation of an innovative training program
Source: Adv Simul (Lond). 2024 May 13;9:18. doi: 10.1186/s41077-024-00289-0 (PMC11092212; doi:10.1186/s41077-024-00289-0)
Supplement: Supplementary file 1 — Supplementary Material 1. [file 41077_2024_289_MOESM1_ESM.zip › Appendix 2_Script for Post-Partum Haemorrhage (PPH) Scenario (in English)_ESM.docx]

**Appendix 2: Script for Post-Partum Haemorrhage (PPH) Scenario (in English)**

| **Scenario theme: Postpartum hemorrhage**  Script editors: A. Chilin / C. Benski | | |
| --- | --- | --- |
| **Goals** | | |
| **Medical**  Diagnose delivery hemorrhage  Recognize hemodynamic emergency  Know the treatment sequence  Find the most common causes  Use uterotonic medications | Managing massive transfusions and crasis disorders  Discuss a course of action (medium-term planning)  **Interprofessional collaboration** |  |
| **Trainers, learners and roles** | | |
| **Debriefer:** Obstetrician  **Facilitators:** 1SF  **Briefing/debriefing:**  **Number of learners and role:** 3 active participants, 2 midwives, 1 obstetrician  **Additional help:** observers, 1 observer: delay, 1 observer: medical care | | |
| **Logistics** | | |
| **Material:**  Bed  Maternal monitoring (BP, pulse, saturation); Snap cuff  Venous lines with tubing and 3-way stopcock  Medications: Oxytocin; Cytotec; Methergine; Nalador ?; Antibiotics: (cefazolin or slow IV ampicillin: 2gr in a single dose)  Infusions: Ringer-Lactate; Sodium chloride 0.9%, Macromolecules  Transfusions: blood: universal donor  O2 mask, urinary catheter  Gloves (to be revised); collar clips; valves; blouse, drapes, compresses  Bakri Ball  Moltex full of blood  Elastic band around the 2nd VVP, remove the band to mimic placement of the 2nd venous line  Newborn doll  Means of communication: telephone images to mime the call  Sheets: Hb/Ht results; Crash; DA/RU; uterus | | |
| **Standardized patient preparation**  Initially patient is calm and collaborative. Answer directed questions. Gradually manifests fatigue and discomfort as the scenario progresses. Loss of consciousness on sign from the Trainer if not adequately supported. | | |
| **Preparation of the accompanist**  Disturbing element: “It bleeds a lot! » “All this blood is normal” “My friend is not feeling well” “I am very worried about my friend”. Can be taken out of the room at the request of the participants. | | |
| **Briefing** | | |
| Welcome, simulation charter presented in the general introduction. | | |
| **Explanation of the session;**  Presentation of the material:   - Show phone photos - Ask to state loud and clear in the event of a call on the telephone: WHO am I calling WHAT degree of urgency….. - Show sheets for certain clinical data at the request of participants (losses; Hb/Ht; soft-hard uterus; results of the three-stage revision)   Explain that there is equipment but that the endo-uterine procedure will not be performed.  There are signs with clinical or biological information that is requested by participants | | |

| **Scenario briefing. Distribution of roles.** |
| --- |
| **Clinical history (to be read as a pre-scenario briefing ONLY to the midwife caring for the patient). The other speakers leave the room**  *We are in the delivery room: we are 45 minutes away from a vaginal birth.*  *Mrs. V, 30-year-old patient, 4G 4P (4 AVB without particularity)*  *For this delivery: admitted for spontaneous labor.*  *The patient quickly gave birth to a child weighing 4450 g with an intact perineum. She received 5 IU of Syntocinon® on the shoulders.*  *Delivery of a placenta 15 minutes after delivery, complete and normal in appearance. Prophylaxis of atony in view of multiparity with Syntocinon 20 U over 6 hours.*  *Improved uterine contraction under this treatment.*  *Total losses estimated at this time at* ***400 ml***  *The midwife leaves the room: the scenario begins*  *45 minutes after delivery the patient reports increased blood loss (“it’s leaking down”) she feels like she’s going to pass out*  *The midwife is called back* |
| **Scénario. Durée : 10 minutes** |
| Scenario start as soon as “**it’s flowing down” is called:**   - **obvious flow (Displays with loss at corresponds to 800 cc)** - **relaxed uterus**   **Displays with BP at 115/80, pulse at 95/min; saturation 98%; FR 13 /** min  Since call   - **DISPLAY: loss 400 cc + 800 cc = 1200 cc** - if palpation of the uterus: **DISPLAY**: SOFT uterus |
| **Evolution of the scenario**  Total HP 1200 cc   - **PUT POSTED** even if no quantity of losses (stress) is requested   **With TA at 95/75, puls. at 110/min; saturation 98%; FR 13 / min; spongy uterus**  Then bleeding another 400 cc   - **DISPLAY: loss 400 cc + 800 cc + 400cc = 1500 cc** - **BP at 70/40 pulse: at 125/min saturation: 98% Fr: 20/min soft uterus**   **Patient with nausea and vomiting (accompanist: “My friend doesn’t feel well, she’s vomiting”)**  **If requested and carried out, put LABORATORY POSTER: Hb 62 g/L; Excl. tax 22%; PTT 42 sec; Fibrinogen 0.9 g/L** |
| **Evolution of the scenario**  Improvement of the situation if correct obstetric care with   - Vascular filling with ringer lactate or NACL 0.9% (1L in 15 minutes) - Placement of a foley catheter - Preparation for a transfusion, check blood availability, determine patient group - Administration of 2nd level uterotonics (Nalador) (+/- Fibrinogen and Cyklokapron) - Uterine massage to expel clots, bi-manual compression if continued heavy bleeding - Revision of the genital tract (check for absence of lesion of the cervix or vagina) with systematic RU, installation for placement of Bakri balloon |
| **After all this:**  **OPTION A**  **BP at 95/75, pulse at 110/min; saturation 98%; FR 13 / min; spongy uterus 0%, bleeding 0%**   - **DISPLAY:** CONTRACTED uterus - **Stopping the scenario (if stabilization of the patient and/or installation for Bakri or planning of embolization)**   **OPTION B**  If suboptimal management: hemodynamic constants remain identical  **BP at 70/40, pulse. at 140/min; saturation 90%; FR 20/min¸ uterus still sluggish; unconscious patient**   - Patient feeling unwell with loss of consciousness - **DISPLAY: unconscious patient** - **Stop the scenario if**   Consideration of transfer to the operating room for hemostasis hysterectomy |

| **Possible observations during the scenario / expected actions** |
| --- |
| **Recognition of post-partum haemorrhage**  close monitoring of vital parameters  uterine massage  bladder emptying / insertion of an indwelling catheter  placental control  3-stage revision (with prophylactic antibiotic therapy)  installation of a 2^e^ large-calibre VVP  infusion of 20 IU Syntocinon® or misoprostol sublingually or methylergometrine IM  blood test request  Administration of fibrinogen / Cyklokapron®.  measure haemoglobin levels  high-flow oxygen therapy if possible  manage blood tests and transfusions if necessary; notify the Transfusion Centre  treatment planning (team considering Bakri or embolisation) |
| **Debriefing** |
| Definition of post-partum haemorrhage: prevention by active delivery management  Difficulty estimating losses  Good early maternal tolerance  Maternal morbidity and mortality (historical)  Risk factors  Most frequent causes (4T: tone; trauma; tissue; thrombin)  Planning, multidisciplinarity and a step-by-step approach   - Suggested algorithm: to be adapted to the local context - Timing: reducing delays in care   Stage 1 (0-30 minutes: timing to be adapted to clinical situation and severity)   - Identifying the aetiology - Mechanical" treatment - Medical resuscitation (call for help / monitoring / venous access / assessment of losses (laboratory) / prevention of hypothermia - Administration of uterotonics (Syntocinon rapid infusion)   Stage 2 (30-60 minutes: timing to be adapted to clinical situation and severity)   - If Syntocinon fails: other uterotonics , Methérgine , Cytotec - Compression / Bakri. Continued filling. - Haemodynamic stabilisation: Transfusion and haemostasis stimulation (fibrinogen / tranexamic acid) - Haemostasis stabilisation goal: Hb>80 g; Plqt > 50G; fibrinogen > 1g/L; TP>50%; pH > 7.2; Temp. >35 °   Stage 3 (>60 minutes: timing to be adapted to clinical situation and severity)   - Planning and anticipating the patient's transfer to a facility for surgery |
