# Supplementary material for: Simulation training on respectful emergency obstetric and neonatal care in north-western Madagascar: a mixed-methods evaluation of an innovative training program
Source: Adv Simul (Lond). 2024 May 13;9:18. doi: 10.1186/s41077-024-00289-0 (PMC11092212; doi:10.1186/s41077-024-00289-0)
Supplement: Supplementary file 1 — Supplementary Material 1. [file 41077_2024_289_MOESM1_ESM.zip › Appendix 3_photos and descriptions _ESM.docx]

**Appendix 3 : photos and descriptions** (NB: All the group gave their oral and written consent.)

**IMG_0775** Title: Participants of group 2 during focus group activity


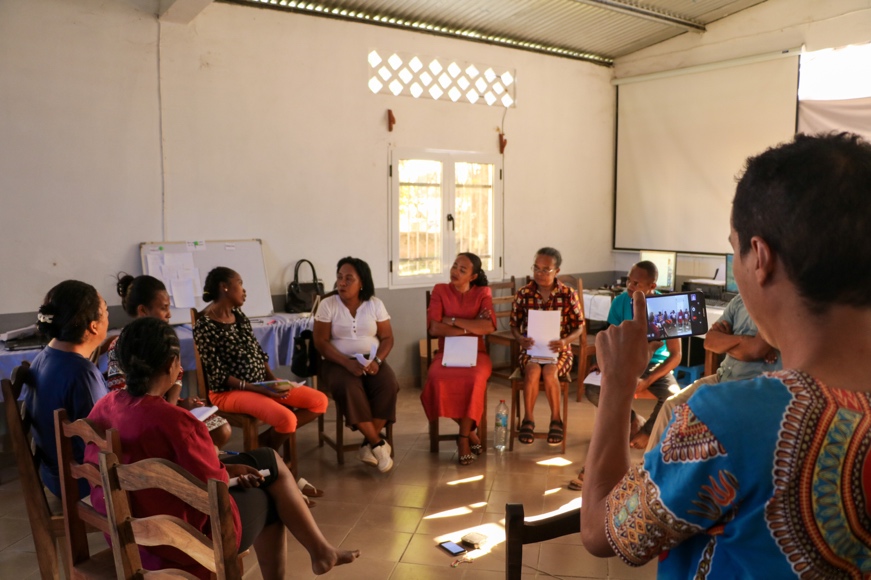
Footer: We can see 5 participants, a coach and 3 facilitators discussing in a circle. The workshop is recorded by video, audio and photos.

**IMG_0665**Title: Exchanges between participants during the focus group activity


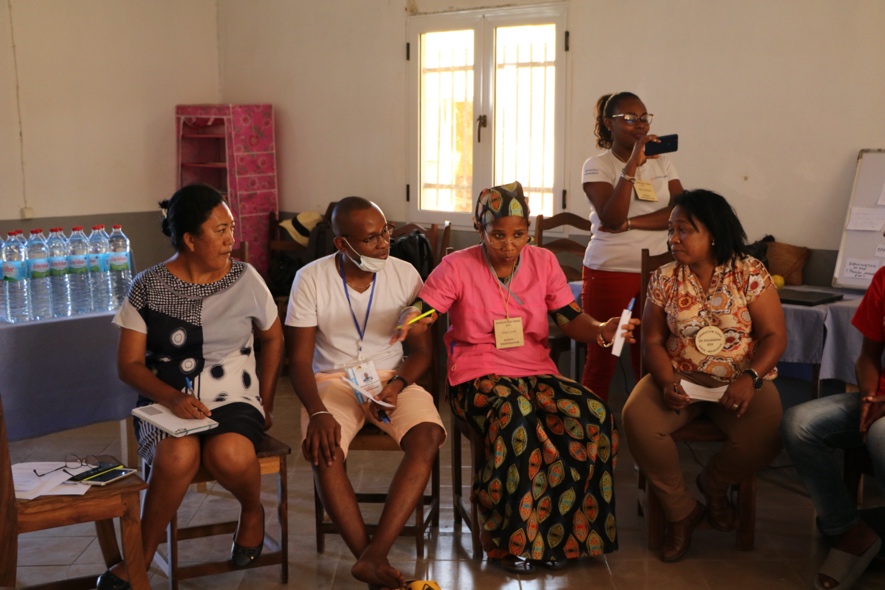


Footer:

Among the 6 participants in group 1, three participants and a facilitator are photographed seated in the middle of a debate with demonstrations of situations encountered during the simulation.

**IMG_0659**Title: Vision board of the post-it” introductory workshop at the start of focus group workshop.


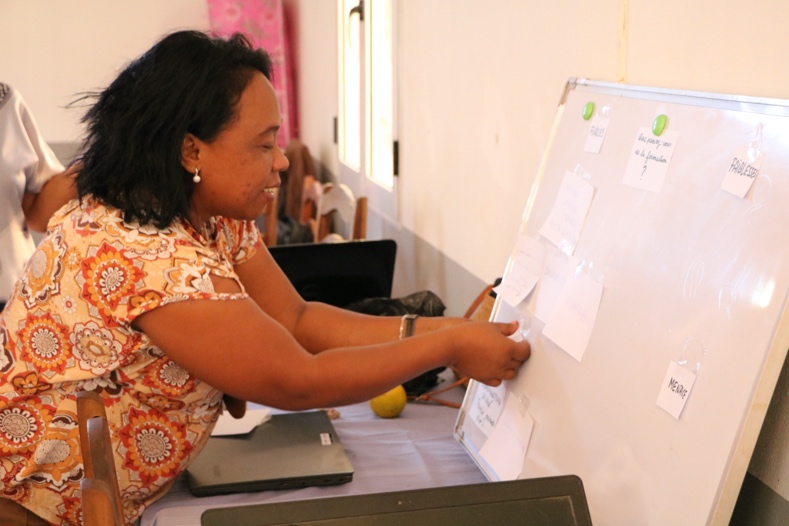


Footer:

Arrangement on a board of words written by the participants during the introductory “post-it” workshop. The participants were invited to give their main feeling regarding the training to begin the focus group discussions.
