# Supplementary figures and images for: Simulation training on respectful emergency obstetric and neonatal care in north-western Madagascar: a mixed-methods evaluation of an innovative training program
Source: Adv Simul (Lond). 2024 May 13;9:18. doi: 10.1186/s41077-024-00289-0 (PMC11092212; doi:10.1186/s41077-024-00289-0)

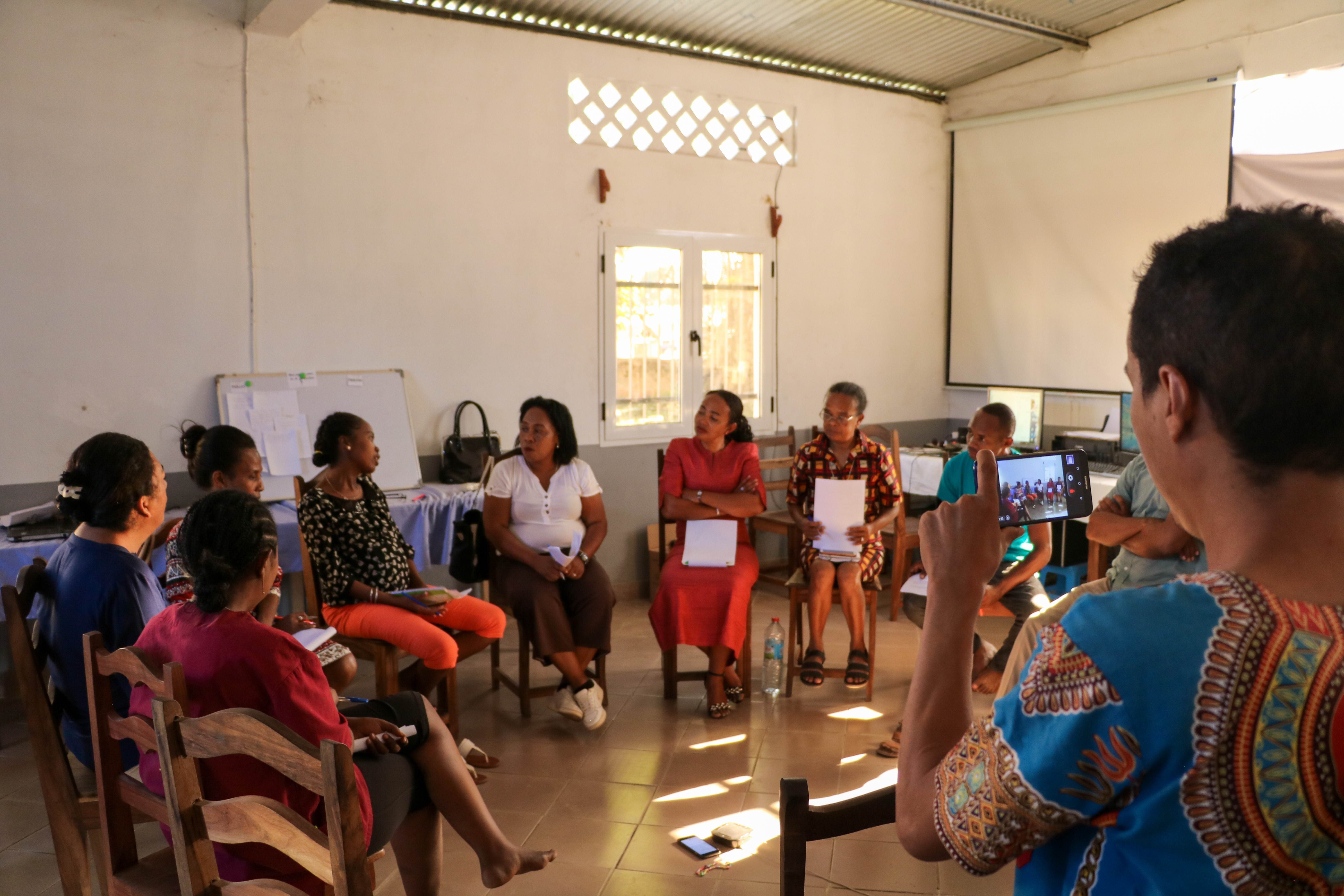

Supplement: Supplementary file 1 — Supplementary Material 1. [file 41077_2024_289_MOESM1_ESM.zip › Photo 1_Participants of group 2 during focus group activity_ESM.JPG]

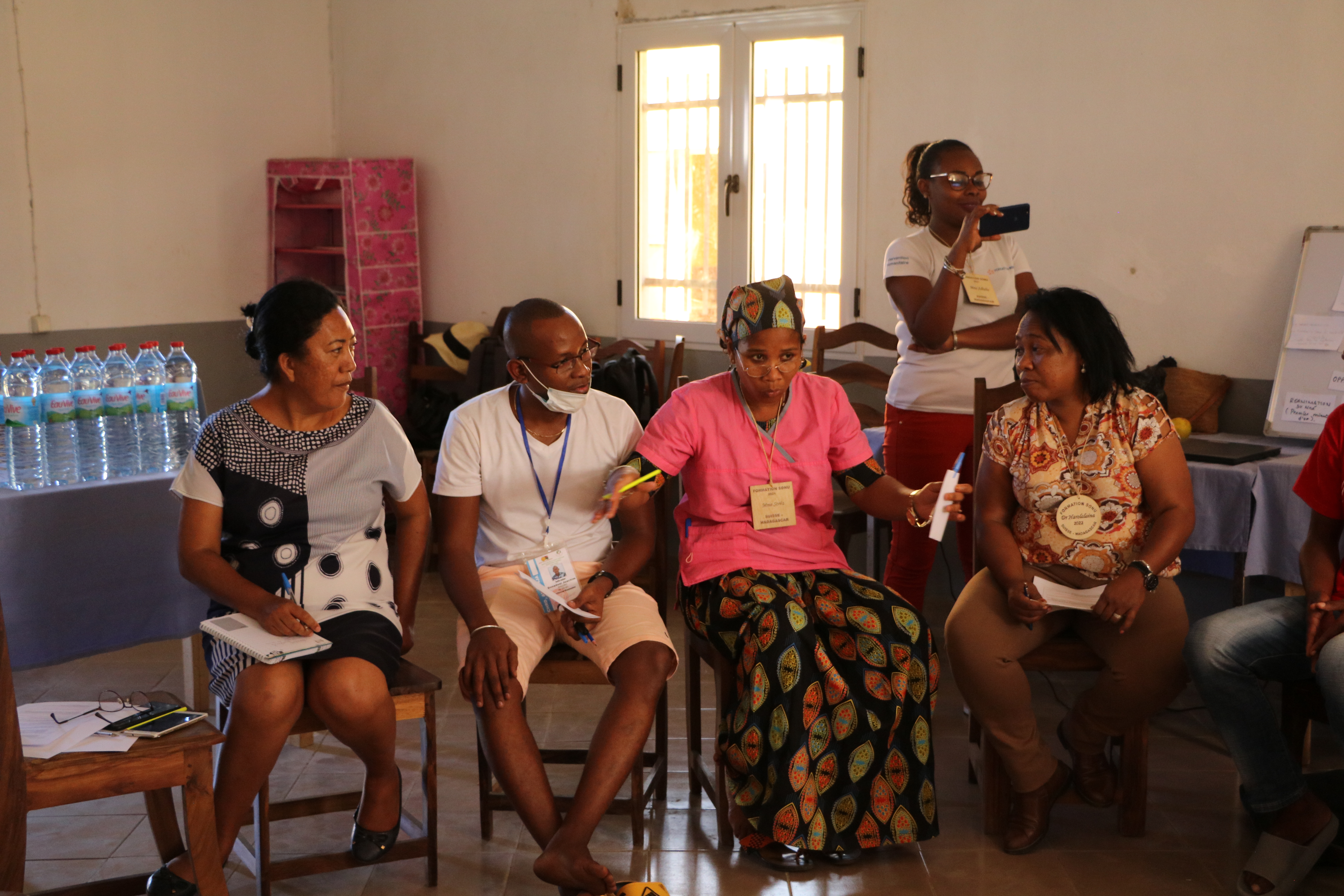

Supplement: Supplementary file 1 — Supplementary Material 1. [file 41077_2024_289_MOESM1_ESM.zip › Photo 2_Exchanges between participants during the focus group activity_ESM.JPG]

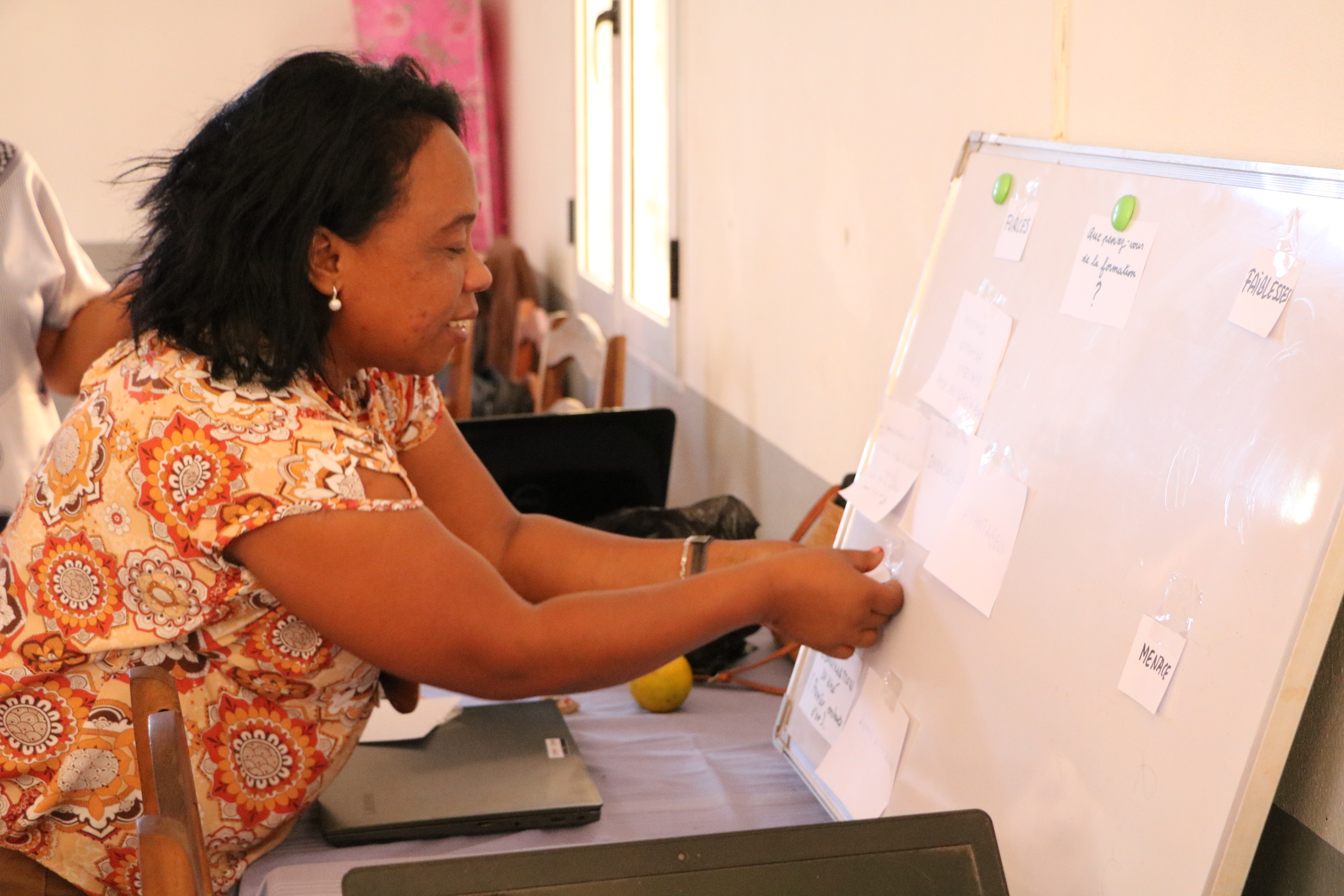

Supplement: Supplementary file 1 — Supplementary Material 1. [file 41077_2024_289_MOESM1_ESM.zip › Photo 3_Vision board of the post-it" introductory workshop at the start of focus group workshop_ESM.JPG]
